# Proportion and factors associated with Hepatitis B viremia in antiretroviral treatment naïve and experienced HIV co-infected Ghanaian patients

**DOI:** 10.1186/s12879-016-1342-4

**Published:** 2016-01-13

**Authors:** Timothy N. A. Archampong, Margaret Lartey, Kwamena W. Sagoe, Adjoa Obo-Akwa, Ernest Kenu, Fizza S. Gillani, Hongmei Yang, Isaac Boamah, Timothy Flanigan, Awewura Kwara

**Affiliations:** 1 Department of Medicine and Therapeutics, School of Medicine and Dentistry, College of Health Sciences, University of Ghana, Accra, Ghana; 2 Korle-Bu Teaching Hospital, Accra, Ghana; 3 Department of Microbiology, School of Biomedical and Allied Health Sciences, College of Health Sciences, University of Ghana, Accra, Ghana; 4 Warren Alpert Medical School of Brown University, Providence, RI USA; 5 The Miriam Hospital, Providence, RI USA; 6 Department of Biostatistics and Computational Biology, University of Rochester School of Medicine and Dentistry, Rochester, NY USA

**Keywords:** ART, HIV and HBV co-infection, Viremia, Sub-Saharan Africa

## Abstract

**Background:**

The global burden of Hepatitis B virus (HBV) and HIV co-infection is enormous. The risk of developing cirrhosis and hepatocellular cancer is associated with HBV DNA levels. The main objective of the study was to determine proportion of Hepatitis B viremia in ART-naïve and ART-experienced co-infected Ghanaian patients and factors associated with HBV viremia after at least 36 weeks of lamivudine with or without tenofovir containing ART.

**Methods:**

Hepatitis B and HIV co-infected patients who were ART-naïve or had received at least 9 months of lamivudine-containing ART were enrolled in a cross-sectional study at Korle-Bu Teaching Hospital. Demographic and clinical data were collected and samples obtained for Hepatitis B serology, liver function tests and HBV DNA. Factors associated with viremia were determined using univariate and multivariate logistic regression analysis.

**Results:**

Of 3108 HIV-infected patients screened, 257 (8.3 %) were HBsAg-positive, of which 235 enrolled. Overall, 152 (64.7 %) were ART-experienced and 83 (35.3 %) were ART-naïve. Eighty-nine-percent of ART-naïve and 42.1 % of ART-experienced patients had HBV DNA > 20 IU/mL. In multivariate analysis of all patients, being ART-naïve (OR 10.1, 95 % CI 4.6 – 21.9) and elevated ALT (OR 3.7, 95 % CI 1.8 – 7.9) were associated with Hepatitis B viremia. In treatment experienced patients, elevated ALT (OR 4.8 CI 2.0 – 12.1) and male sex (OR 2.1, 95 % CI 1.0 – 4.2) were associated with Hepatitis B viremia.

**Conclusions:**

Majority of ART-naïve (89 %) and 42 % of ART-experienced patients had detectable hepatitis B viremia > 20 IU/mL. An abnormal serum ALT was significantly associated with hepatitis B viremia in HBV and HIV co-infected patients irrespective of treatment status. Baseline and on-treatment ALT may be a useful non-invasive predictor of Hepatitis B viremia in resource-constrained countries in sub-Saharan Africa where infection is endemic and viral load tests are not widely available.

## Background

Globally, it is estimated that there are 240 million persons chronically infected with HBV infection, with a majority of them living in low- and middle-income countries [1]. The prevalence of chronic HBV infection varies from 0.1 – 2 % in industrialized countries to 5 – 10 % in Sub-Saharan Africa [1]. An estimated 6 to 14 % of HIV-infected persons worldwide are chronically infected with HBV [2], with the highest rates occurring in sub-Saharan Africa and Asia [3]. The natural history of chronic HBV is negatively affected by HIV co-infection. HBV/HIV co-infected patients have lower spontaneous HBeAg seroconversion rates, higher levels of HBV viremia [4], faster progression to cirrhosis and increased rate of development of HCC [5].

The risk of developing cirrhosis, hepatic decompensation and HCC is associated with HBV DNA levels [6, 7]. HIV viremia is also associated with progression of HBV infection [5]. Thus, suppression of HBV DNA and HIV RNA to undetectable levels is an important treatment goal of HIV/HBV-co-infected patients [4, 8, 9]. In treated HIV/HBV co-infected patients, detectable HBV viremia has been associated with positive HBeAg, negative anti-HBe, HBV genotype A, type and duration of ART, CD4 count < 200 cells/mm3, detectable HIV RNA, and <95 % adherence to ART [10].

In Ghana, the reported prevalence of HBV ranges from 12 to 15 % among blood donors [11]. At the Fevers Unit, Korle-Bu Teaching Hospital (KBTH), an earlier study of 154 HIV patients found the prevalence of HBV infection was 13 % [12]. In another study in Kumasi, Ghana, the prevalence of HBV infection in a cohort of 838 HIV-infected patients was found to be 16.7 % [13]. Although HIV patients are increasingly screened for HbsAg, HBV DNA testing is not routinely done in Ghana due to cost implications. Until tenofovir became available in Ghana, the recommendation was to use lamivudine (3TC)-containing ART for HBV/HIV co-infected persons. The most recent HIV treatment guidelines recommend the use of tenofovir (TDF) plus 3TC or emtricitabine in patients with HBV/HBV co-infection [14]. The HIV treatment guidelines also recommend using HBV viral load (where available) and ALT for treatment initiation and monitoring. The objective of this study was to determine the proportion of HBV viremia in ART-naïve and treatment-experienced HIV co-infected Ghanaian patients, and to investigate the factors associated with viremia after at least 9 months of lamivudine with or without tenofovir containing ART.

## Methods

### Study participants and study design

Korle-Bu Teaching Hospital has 2500 beds and is the main tertiary referral centre in Accra serving the majority of the southern half of Ghana. The Fevers Unit is the principal referral centre for infectious diseases in Southern Ghana with approximately 15,000 HIV patients under regular follow-up in the out-patient clinic and over 7000 patients on regular anti-retroviral therapy. It has an in-patient department staffed with doctors, nurses, counselors, para-clinical and laboratory staff which offers service to a wide range of patients with infectious diseases including HIV.

This study used a cross-sectional design to recruit HBV-HIV co-infected patients attending the Fevers (Infectious Diseases) Unit, Korle-bu, Accra, Ghana between 2012 and 2014. Adult HIV patients attending the Fevers (Infectious Diseases) Unit during the study period (2012–2014) were screened for HBsAg using the Core HBsAg rapid test (Core Diagnostics, Birmingham, UK) following informed consent. HIV and HBsAg-positive study participants were enrolled into two groups. The treatment-naive group (t0) were HBV-HIV co-infected patients who had not initiated therapy. The treatment-experienced group (t1) were HBV-HIV co-infected patients who had been on at least 9 months of 3TC containing anti-retroviral therapy. Patients were excluded if they declined to sign a consent form for participation. Demographic and clinical data were collected from enrolled patients using standardized data collection form. Medical records were also reviewed where additional clinical and laboratory data including CD4 cell count extracted as patients did not know their most recent CD4 count. Adherence status was determined using the validated Simplified Medication Adherence Questionnaire (SMAQ) [15]. All extracted data was subsequently entered into Microsoft Access.

The Institutional Review Board (IRB) of the University of Ghana Medical School, Accra, Ghana and Lifespan Hospitals, Providence, Rhode Island reviewed and approved the study.

### Laboratory analysis

Enrolled patients had blood drawn into an EDTA and serum separating tube (SST). Blood in EDTA was transported to the Virology Laboratory, Korle-Bu, on ice, centrifuged within 4 h of collection using EBA 20 table-top centrifuge (Hettich Zentrifugen, Tuttlingen, Germany), and plasma frozen at −80 in ~ 600 ul aliquots. HBsAg positivity was confirmed serologically using a third generation ELISA Surase B 96 (TMB) (General Biologicals Corp, Hsinchu, Taiwan). Plasma was also tested for Hepatitis B e-antigen (HBeAg), Hepatitis B e-antibody (anti-HBe) using EASE BN-96 (TMB) and Hepatitis B-core antibody (anti-HBc) using ANTICORASE MB-96 (TMB) from General Biologicals, Hsinchu, Taiwan. Blood in SST was tested for liver function. Plasma HBV DNA was determined using fully automated COBAS® TaqMan® Analyzer (Roche Diagnostics GmbH, Mannheim, Germany). The lower limit of detection of the Roche TaqMan assay was 20 IU mL-1 and the linear range 20 – 170,000,000 IU/mL.

### Statistical analysis

Statistical analyses were performed using Software SAS 9.3 (SAS Institute Inc, Cary, NC). Bivariate analyses of association between participant factors and treatment status or HBV viremia were assessed by Student’s *t*-test for continuous variables or Chi-square test for categorical variables. Logistic regression with variable selection by stepwise was used to find the joint effect of the participants (treatment status, age, gender, BMI, CD4 counts, serum ALT, AST and ALB) on HBV viremia using all the data and also using treatment-specific data, and the findings were confirmed by the Smoothly Clipped Absolute Deviation (SCAD) method [16, 17]. For all analysis, a *P* value < 0.05 was considered significant.

## Results

### Study population and baseline characteristics

During the study period, 3108 HIV-infected patients were screened for HBV with HBsAg, of which 257 patients (8.3 %) were HBsAg positive. Two hundred and thirty-five (235) patients were recruited into the study, with 22 patients either declining consent or had incomplete data. The baseline characteristics of all patients and by ART status are shown in Table 1. Overall, 152 (64.7 %) of the patients were treatment-experienced and 83 (35.3 %) were treatment-naïve. In treatment-naïve and treatment experienced patients, the median HBV DNA log_10_IU/mL was 8.8 (IQR: 4.8–17.6) and 2.9 (IQR: 2.1–6.8) respectively. The mean (SD) duration of ART in treatment-experienced patients was 4.2 (2.7) years. Treatment-experienced and naïve patients were similar except that treatment-experienced patients were older, had higher mean weight, higher mean BMI and higher mean CD4 count Table 1. The ART-experienced patients were less likely to have serum albumin level below the lower limit of normal or AST, ALT, and HBV DNA levels above the upper limit of normal (ULN), Table 1.

**Table 1:** Baseline characteristics of HIV/HBV co-infected patients and by antiretroviral treatment Status

		Treatment status			
Characteristic	All patients (*N* = 235)	Experienced (*N* = 152)	Naïve (*N* = 83)	*P* value *T* test	*P* value Wilcoxon rank sum test
Mean (SD) age (years)	41.0 (10.0)	42.6 (9.5)	37.9 (10.2)	0.0006	<0.0001
Mean (SD) weight (kg)	63.1 (13.4)	64.9 (13.1)	59.6 (13.2)	0.0038	0.0082
Mean (SD) BMI (kg/m2)	23.4 (4.7)	24.2 (4.7)	21.8 (4.5)	0.0001	0.0002
Mean (SD) CD4 count (cells/uL)	460.7 (322)	513 (304)	363 (334)	0.0006	<0.0001
	n (%)	n (%)	n (%)	*P* value Chi square	*P* value Fisher
Sex				0.597	0.677
Female	139 (59.1)	88 (57.9)	51 (61.5)		
Male	96 (40.9)	64 (42.1)	32 (38.6)		
Ethnicity				0.744	0.762
Akan	109 (46.8)	72 (47.4)	37 (45.7)		
Ewe	44 (18.9)	29 (19.1)	15 (18.5)		
Ga	28 (12.0)	20 (13.1)	8 (9.9)		
Other	52 (22.3)	31 (20.4)	21 (25.9)		
WHO HIV stage				0.630	0.619
1	55 (23.7)	38 (25.3)	17 (20.7)		
2	58 (25.0)	36 (24.0)	22 (26.8)		
3	92 (39.7)	61 (40.7)	31 (37.8)		
4	27 (11.6)	15 (10.0)	12 (14.6)		
HBeAg positive	47 (20.0)	28 (18.4)	19 (22.9)	0.413	0.495
HBeAb positive	188 (80.0)	122 (80.3)	66 (79.5)	0.891	1.000
HBeAg/eAb combination				0.622	0.604
HBeAg positive/Ab negative	41 (17.5)	25 (16.5)	16 (19.3)		
HBeAg negative/Ab positive	182 (77.5)	119 (78.3)	63 (75.9)		
HBeAg negative/Ab negative	6 (2.6)	5 (3.3)	1 (1.2)		
HBcIgM positive	5 (2.1)	4 (2.6)	1 (1.2)	0.469	0.659
		Treatment status			
Characteristic	All patients	Experienced	Naïve	*P* value Chi square	*P* value Fisher
	*N* = 235 (%)	(*N* = 152)	(*N* = 83)		
		n (%)	n (%)		
Alanine aminotransferase				0.0012	0.0015
Normal (5-40 U/L)	169 (71.9)	120 (79.0)	49 (59.0)		
Elevated > ULN	66 (28.1)	32 (21.1)	34 (41.0)		
Aspartate aminotransferase				0.0005	0.0008
Normal (5-50 U/L)	178 (75.7)	126 (82.9)	52 (62.7)		
Elevated > ULN	57 (24.3)	26 (17.1)	30 (36.1)		
Albumin				<0.0001	<0.0001
Normal (36-50)	153 (65.1)	117 (77.0)	36 (43.4)		
Low < LLN	82 (34.9)	35 (23.0)	47 (56.6)		
HBV DNA (IU mL-1)				<0.0001	<0.0001
< 20	97 (41.3)	88 (57.9)	9 (10.8)		
20 – 2000	58 (24.7)	31 (20.4)	27 (32.5)		
2001 - 20, 000	14 (6.0)	4 (2.6)	10 (12.1)		
> 20, 000	66 (28.1)	29 (19.1)	37 (44.6)		
Initial ART contain					
3TC	100 (42.6)	100 (65.8)	--		
3TC + TDF	52 (22.1)	52 (34.2)	--		
Duration on ART			--		
<1 year	4 (1.7)	4 (2.6)			
1 – 2 years	38 (16.2)	38 (25.0)	--		
2 – 3 years	21 (8.9)	21 (13.8)	--		
> 3 years	89 (37.9)	89 (58.6)	--		
Ever forgotten meds?		36 (23.7)	--		
Do you take meds on time?		16 (10.5)	--		
Stop taking meds due to illness?		12 (7.9)	--		
Forgotten meds last weekend?		14 (9.2)	--		
Number of times not taken meds last week?					
0		57 (37.5)			
1 to 2		10 (6.6)			
3 – 5		6 (3.9)			
more than 5		3 (2.0)			

*BMI* body mass index

*HBeAg/Ab* Hepatitis B e Antigen/Antibody

*HBcIgM* Hepatitis B core IgM

*3TC* lamivudine

*TDF* tenofovir

*ULN* upper limit of normal

*LLN* lower limit of normal

*ART* anti-retroviral therapy

Of the 152 treatment-experienced patients, 100 (65.8 %) were on ART with 3TC (3TC), while 52 had received 3TC + TDF containing ART, for at least 9 months prior to sampling (3TC + TDF). Of the (3TC + TDF) group, 37 (71.2 %) were on combination therapy from the onset of treatment while 15 (28.8 %) had been on another 3TC containing regimen before changing to 3TC + TDF combination therapy.

In the 3TC-only group, the median duration of treatment was 4.85 years (IQR: 3.0–7.1). In the 3TC + TDF group, median duration of treatment of patients on 3TC and TDF from the onset was 1.23 years (IQR: 1.1–1.6) while 4.87 years (IQR: 3.8–5.9) in patients on another 3TC regimen before changing to 3TC + TDF combination therapy.

Overall, 74 (89.2 %) of the 83 ART-naïve and 64 (42.1 %) of the 152 ART-experienced patients had HBV DNA > 20 IU/mL (Table 1). Forty-seven (56.6 %) of ART-naïve and 33 (21.7 %) of ART-experienced patients had HBV DNA > 2000 IU/mL.

### Factors associated with hepatitis B viremia

Bivariate analysis of factors associated with viremia (HBV DNA > 20 IU/mL) in the treatment-naïve and experienced patients are shown in Tables 2 and 3. Males were more likely to have detectable HBV DNA (>20 IU/mL) in comparison to females during ART (53 % vs 47 %, *P* = 0.019). An elevated serum AST and ALT above ULN were also more likely to be associated with detectable HBV during ART (*P* = 0.0004) and (*P* = 0.0001), respectively. In treated patients, being HBeAg-positive and HBeAb-negative was significantly associated with detectable HBV DNA (*P* < 0.0001). Treatment regimens (3TC) and (3TC + TDF) had similar likelihood of hepatitis B DNA suppression, with undetectable Hepatitis B virus in 42.0 and 42.3 % respectively (*P* = 0.97). Of the 83 treatment-naïve patients, only 9 (10.8 %) had HBV DNA < 20 IU/mL.

**Table 2:** Characteristics of antiretroviral treatment-experienced HIV/HBV Co-infected patients with undetectable HBVDNA compared to those with detectable HBV DNA

Characteristic	HBV DNA < 20	HBV DNA ≥ 20	*P*-value *T* test	*P*-value Wilcoxon rank sum test
	IU/mL (*N* = 64)	IU/mL (*N* = 88)		
Mean (SD) age (years)	42.6 (9.6)	42.7 (9.5)	0.928	0.701
Mean (SD) weight (kg)	65.9 (13.7)	63.5 (12.2)	0.262	0.326
Mean (SD) BMI (kg/m2)	24.5 (4.6)	23.9 (4.7)	0.415	0.432
Mean (SD) CD4 count (cells/uL)	539 (257)	478 (358)	0.244	0.157
	n (%)	n (%)	*P* value Chi square	*P* value Fisher
Male sex	30 (46.9 %)	34 (53.1 %)	0.019	0.021
Ethnicity			0.656	0.679
Akan	43 (59.7 %)	29 (40.3 %)		
Ewe	14 (48.3 %)	15 (51.7 %)		
Ga	13 (65.0 %)	7 (35.0 %)		
Other	18 (58.1 %)	13 (41.9 %)		
WHO HIV stage			0.086	0.095
1	24 (63.2 %)	14 (36.8 %)		
2	21 (58.3 %)	15 (41.7 %)		
3	37 (60.7 %)	24 (39.3 %)		
4	4 (26.7 %)	11 (73.3 %)		
HBeAg positive	0	28 (100.0 %)	<0.0001	<0.0001
HBeAb positive	85 (69.7 %)	37 (30.3 %)	<0.0001	<0.0001
HB eAg/E Ab combination			<0.0001	<0.0001
HBeAg positive/Ab negative	0	25 (100.0 %)		
HBeAg negative/Ab positive	85 (71.4 %)	34 (28.6 %)		
HBeAg negative/Ab negative	3 (60.0 %)	2 (40.0 %)		
HBeAg positive/Ab positive	0	3 (100.0 %)		
HBcIgM positive	0	4 (100.0 %)	0.0175	0.030
Alanine aminotransferase			0.0001	0.0002
Normal (5-40 U/L)	79 (65.8 %)	41 (34.2 %)		
Elevated > ULN	9 (28.1 %)	23 (71.9 %)		
Aspartate aminotransferase			0.0004	0.0008
Normal (5-50 U/L)	81 (64.3 %)	45 (35.7 %)		
Elevated > ULN	7 (26.9 %)	19 (73.1 %)		
Characteristic	HBV DNA < 20 IU mL-1	HBV DNA ≥ 20 IU mL-1	*P* value Chi square	*P* value Fisher
	n (%)	n (%)		
Albumin			0.096	0.119
Normal (36-50 g/L)	72 (61.5 %)	45 (38.5 %)		
Low < LLN	16 (45.7 %)	19 (54.3 %)		
Initial ART contain			0.971	1.000
3TC (*n* = 100)	42 (42.0 %)	58 (58.0 %)		
3TC + TDF (*n* = 52)	22 (42.3 %)	30 (57.7 %)		
Duration on ART			0.969	0.972
<1 year	3 (75.0 %)	1 (25.0 %)		
1 – 2 years	22 (57.9 %)	16 (42.1 %)		
2 – 3 years	12 (57.1 %)	9 (42.9 %)		
> 3 years	51 (57.3 %)	38 (42.7 %)		
Ever forgotten meds?	26 (72.2 %)	10 (27.8 %)	0.046	
Do you take meds on time?	8 (50.0 %)	8 (50.0 %)	0.499	
Stop taking meds due to illness?	9 (75.0 %)	3 (25.0 %)	0.211	
Forgotten meds last weekend?	10 (71.4 %)	4 (28.6 %)	0.282	
Number of times not taken meds last week?			--	
0	35 (61.4 %)	22 (38.6 %)		
1 - 2	7 (70.0 %)	3 (30.0 %)		
3 – 5	4 (66.7 %)	2 (33.3 %)		
more than 5	1 (33.3 %)	2 (66.7 %)		

*BMI* body mass index

*HBeAg/Ab* Hepatitis B e Antigen/Antibody

*HBcIgM* Hepatitis B core IgM

*3TC* lamivudine

*TDF* tenofovir

*ULN* upper limit of normal

*LLN* lower limit of normal

*ART* anti-retroviral therapy

**Table 3:** Characteristics of antiretroviral treatment-naive HIV/HBV Co-infected patients with undetectable HBVDNA compared to those with detectable HBV DNA

Characteristic	HBV DNA < 20 IU/mL	HBV DNA ≥ 20 IU/mL	*P*-value *T* test	*P*-value Wilcoxon rank sum test
	(*N* = 64)	(*N* = 88)		
Mean (SD) age (years)	39.6 (8.0)	37.7 (10.4)	0.616	0.288
Mean (SD) weight (kg)	66.2 (9.5)	58.8 (13.4)	0.116	0.092
Mean (SD) BMI (kg/m2)	23.7 (4.9)	21.6 (4.4)	0.184	0.209
Mean (SD) CD4 count (cells/uL)	561.7 (504.5)	338.8 (302.7)	0.228	0.144
	n (%)	n (%)	*P*-value Chi square	*P*-value Fisher
Male sex	4 (12.5 %)	28 (87.5 %)	0.701	0.728
Ethnicity			0.404	0.381
Akan	6 (16.2 %)	31 (83.8 %)		
Ewe	0 (0.0 %)	15 (100.0 %)		
Ga	1 (12.5 %)	7 (87.5 %)		
Other	2 (9.5 %)	19 (90.5 %)		
WHO HIV stage			0.265	0.174
1 & 2	2 (5.1 %)	37 (94.9 %)		
3 & 4	7 (16.3 %)	36 (83.7 %)		
HBeAg positive	0 (0.0 %)	19 (100.0 %)	0.083	0.110
HBeAb positive	9 (13.6 %)	57 (86.4 %)	0.107	0.193
HBeAg/E Ab combination			0.361	0.339
HBeAg positive/Ab negative	0 (0.0 %)	16 (100.0 %)		
HBeAg negative/Ab positive	9 (14.3 %)	54 (85.7 %)		
HBeAg negative/Ab negative	0 (0.0 %)	1 (100.0 %)		
HBeAg positive/Ab positive	0 (0.0 %)	3 (100.0 %)		
HBcIgM positive	0 (0.0 %)	1 (100.0 %)	0.726	1.000
Alanine aminotransferase			0.622	0.731
Normal (5–40 U/L)	6 (12.2 %)	43 (87.8 %)		
Elevated > ULN	3 (8.8 %)	31 (91.2 %)		
Aspartate aminotransferase			0.320	0.473
Normal (5–50 U/L)	7 (13.5 %)	45 (86.5 %)		
Elevated > ULN	2 (6.5 %)	29 (93.6 %)		
Albumin			0.945	1.000
Normal (36–50 g/L)	4 (11.1 %)	42 (89.4 %)		
Low < LLN	5 (10.6 %)	19 (54.3 %)		

*BMI* body mass index

*HBeAg/Ab* Hepatitis B e Antigen/Antibody

*HBcIgM* Hepatitis B core IgM

*ULN*: upper limit of normal

*LLN* lower limit of normal

### Factors associated with viremia in multivariate analysis

In multivariate analysis of all patients, naïve patients were more likely than the treatment-experienced group to have detectable HBV DNA (OR 10.1, 95 % CI 4.6 – 21.9). Additionally, elevated serum ALT level above ULN compared with normal ALT was associated with 3.7-fold increase in likelihood of detectable HBV DNA (OR 3.7, 95 % CI 1.8 – 7.9). Among the treatment-experienced patients, elevated serum ALT level above ULN compared with normal ALT (OR 4.8, CI 2.1-12.1) and Male sex compared Female (OR 2.1, 95 % CI 1.0 – 4.2) were more likely to have detectable HBV DNA (Table 4). Multivariate analysis of treatment-naïve patients did not yield any significant results due to small sample size.

**Table 4:** Factors associated with detectable HBV DNA in multivariate model in all patients and antiretroviral treatment-experienced

Factor	All patients			ART-experienced		
	Odds ratio	95 % Confidence Interval	*P* value	Odds ratio	95 % Confidence Interval	*P* value
Sex (Male vs Female)	--	--	--	2.1	1.0 – 4.2	0.0320
ART (Naïve vs. Experienced)	10.1	4.6 – 21.9	<0.0001	--	--	--
ALT (Elevated vs. Normal)	3.7	1.8 – 7.9	0.0006	4.8	2.1 – 12.1	0.0001
ALB (low vs. normal)	--	--	--	1.9	0.8 – 4.2	0.1326

*ART* anti-retroviral therapy

*ALT* serum Alanine aminotransferase

*ALB*: serum albumin

## Discussion

In this cross-sectional study, we found the prevalence of detectable HBV DNA ≥ 20 IU/mL in HIV-infected patients who were HBsAg positive to be 89.1 % in ART-naïve and 42.1 % in patients on lamivudine containing therapy with or without tenofovir for an average duration of 4.2 years (range, 9 months to 10.4 years). In all patients, ART status and serum ALT level above ULN were associated with HBV viremia. Among the treatment-experienced patients, male sex and ALT level above ULN were associated with Hepatitis B viremia in both univariate and multivariate analysis. Earlier guidelines recommend that HBV DNA be fully suppressed by 12 months of TDF-based therapy in Hepatitis B-HIV infected patients [8, 18]. However, incomplete HBV DNA suppression has been shown to occur in 54 % following 12 months of TDF-based ART [8].

A longer duration of TDF therapy is required for achievement of virologic response (HBV DNA < 20 IU/mL) in co-infected patients. Among HBeAg-positive patients, 92 % (*n* = 67) had HBV DNA < 20 IU/mL after 5 years of treatment [19]. In HBeAg-negative patients, 100 % (*n* = 15) achieved undetectable HBV DNA in 4 years [19]. Similarly, after a median of 74.7 months, 86.5 % (*n* = 111) of co-infected patients demonstrated a virologic response with HBV DNA < 60 IU/mL [20]. There was no difference between patients with or without 3TC [19]. Tenofovir-based ART therefore achieves high HBV DNA suppression ~90 % at 5 years without evidence of resistance in comparison to 3TC only ART [21].

In our study, HBV DNA was undetectable in 42.0 and 42.3 % irrespective of whether TDF was included in the regimen. The median duration on therapy and IQR for 3TC-only and 3TC + TDF were 4.85 years IQR (3.02–7.16) and 4.87 years IQR (3.81–5.85) respectively. This suggests the influence of other factors in sub-optimal therapeutic response. Poor adherence is often considered as an important factor in patients with persistently detectable HBV DNA on nucleoside analogue therapy [20]. It is also possible that the 33 (21.7 %), treated patients with HBV DNA > 2000 IU/mL harbor HBV resistant mutants. After a median of 45 months of 3TC, HBV DNA was found to be undetectable (<14 IU/mL) in 49.1 % (*n* = 106) of co-infected patients in Kumasi, Ghana [22]. Among patients with detectable HBV DNA, 42.6 % also had detectable HIV RNA [22]. Overall, 29.6 % of patients harbored one or more 3TC resistance mutations comprising M204I or M24V in all cases [22].

In this study, HIV RNA was not determined. Its cross-sectional design also did not allow for a temporal assessment of the influence of study predictors such as pre-treatment HBV DNA, adherence on virologic response in treated patients. Furthermore, the small sample size of treatment naive patients limited the evaluation of predictors and detectable HBV DNA.

The serum aminotransferases are sensitive indicators of liver cell inflammation [23]. The most commonly measured are ALT and AST. In HBV-infection, high ALT levels > 30 IU/L in males and 19 IU/L in females, HBV DNA > 20,000 IU/mL can better differentiate between active chronic HBV patients from inactive chronic carriers [24]. However, an increase in serum ALT in co-infected patients may be the result of ART hepatotoxicity, drug resistance, immune reconstitution, drug withdrawal, anti-tuberculous therapy and opportunistic or super-infections [21, 25]. In our study, an elevated ALT was significantly associated with viremia in HBV/HIV co-infected patients irrespective of treatment status. In comparison with HBeAg status, the relationship between serum ALT and viremia in treated patients persisted following multivariate analysis.

We found that males had higher baseline HBV DNA in comparison to females in naïve and ART-experienced patients. The reason is unclear, and will be in keeping with an increased liver-related mortality rate in men with HIV and HBsAg compared with those with only HBsAg (14.2/1000 compared with 0.8/1000) [26]. Reviews in Africa have shown that women are more likely to access and maintain follow up for HIV care [27, 28]. Additionally, in co-infected patients, women had higher intracellular trough concentrations of TDF in a pilot pharmacokinetic study [29]. This raises the possibility of gender differences in adherence or intracellular concentrations of the nucleoside reverse transcriptase inhibitors diphosphate or triphosphates, which would need evaluation in prospective studies.

## Conclusion

Routine Hepatitis B screening is essential in the evaluation and management of HIV patients. Elevated HBV DNA level is associated with overall mortality in HBV/HIV co-infected patients, especially during the period before development of AIDS [30], and it is essential to identify patients who have viremia for treatment intensification. Majority of ART-naïve (89 %) and 42 % of ART-experienced patients were found to have detectable HBV. An abnormal serum ALT was significantly associated with HBV viremia in HBV-HIV co-infected patients irrespective of treatment status. Baseline and on-treatment ALT may therefore be a useful non-invasive cost-effective predictor of HBV viremia in resource-constrained countries in sub-Saharan Africa where the infection is endemic thereby assisting in risk stratification of patients for liver-related complications. Serum ALT may serve as a useful tool on monitoring disease activity and treatment response in naïve and ART-experienced patients and could be used to prioritize when or which patients should get more costly viral load testing to aid decision about optimizing treatment.

## Abbreviations

3TC: Lamivudine
ALB: serum albumin
ALT: serum alanine Aminotransferase
ART: Anti-retroviral therapy
AST: serum aspartate Aminotransferase
BMI: Body Mass Index
HBcIgG: Hepatitis B-c-IgG
HBcIgM: Hepatitis B-c-IgM
HBeAb: Hepatitis B-e-Antibody
HBeAg: Hepatitis B-e-Antigen
HBsAg: Hepatitis B-surface Antigen
HBV: Hepatitis B
HCC: Hepatocellular carcinoma
HIV: Human Immunodeficiency Virus
IQR: Inter-quartile range
KBTH: Korle-Bu Teaching Hospital
TDF: Tenofovir
ULN: Upper limit of normal
